# Medullary thick ascending limb impairment in the *Gla^tm^Tg(CAG-A4GALT)* Fabry model mice

**DOI:** 10.1096/fj.201701374R

**Published:** 2018-03-19

**Authors:** Hiroki Maruyama, Atsumi Taguchi, Yuji Nishikawa, Chu Guili, Mariko Mikame, Masaaki Nameta, Yutaka Yamaguchi, Mitsuhiro Ueno, Naofumi Imai, Yumi Ito, Takahiko Nakagawa, Ichiei Narita, Satoshi Ishii

**Affiliations:** * Department of Clinical Nephroscience, Niigata University Graduate School of Medical and Dental Sciences, Niigata, Japan;; † Department of Matrix Medicine, Faculty of Medicine, Oita University, Yufu, Japan;; ‡ Division of Tumor Pathology, Department of Pathology, Asahikawa Medical University, Asahikawa, Japan;; § Electron Microscope Core Facility, Niigata University, Niigata, Japan;; ¶ Yamaguchi’s Pathology Laboratory, Matsudo, Japan;; ‖ University Health Center, Joetsu University of Education, Joetsu, Japan;; # Division of Clinical Nephrology and Rheumatology, Niigata University Graduate School of Medical and Dental Sciences, Niigata, Japan;; ** Department of Health Promotion Medicine, Niigata University Graduate School of Medical and Dental Sciences, Niigata, Japan;; †† Future Basic Medicine, Nara Medical University, Kashihara, Japan;; ‡‡ GlycoPharma Corporation, Oita, Japan

**Keywords:** polyuria, mitochondria-rich cell, uromodulin, Na^+^-K^+^-2Cl^−^ cotransporter, Na^+^-K^+^-ATPase

## Abstract

A main feature of Fabry disease is nephropathy, with polyuria an early manifestation; however, the mechanism that underlies polyuria and affected tubules is unknown. To increase globotriaosylceramide (Gb3) levels, we previously crossbred asymptomatic *Gla^tm^* mice with transgenic mice that expressed human Gb3 synthase (A4GALT) and generated the *Gla^tm^Tg(CAG-A4GALT)* symptomatic Fabry model mice. Additional analyses revealed that these mice exhibit polyuria and renal dysfunction without remarkable glomerular damage. In the present study, we investigated the mechanism of polyuria and renal dysfunction in these mice. Gb3 accumulation was mostly detected in the medulla; medullary thick ascending limbs (mTALs) were the most vacuolated tubules. mTAL cells contained lamellar bodies and had lost their characteristic structure (*i.e*., extensive infolding and numerous elongated mitochondria). Decreased expression of the major molecules—Na^+^-K^+^-ATPase, uromodulin, and Na^+^-K^+^-2Cl^−^ cotransporter—that are involved in Na^+^ reabsorption in mTALs and the associated loss of urine-concentrating ability resulted in progressive water- and salt-loss phenotypes. *Gla^tm^Tg(CAG-A4GALT)* mice exhibited fibrosis around mTALs and renal dysfunction. These and other features were consistent with pathologic findings in patients with Fabry disease. Results demonstrate that mTAL dysfunction causes polyuria and renal impairment and contributes to the pathophysiology of Fabry nephropathy.—Maruyama, H., Taguchi, A., Nishikawa, Y., Guili, C., Mikame, M., Nameta, M., Yamaguchi, Y., Ueno, M., Imai, N., Ito, Y., Nakagawa, T., Narita, I., Ishii, S. Medullary thick ascending limb impairment in the *Gla^tm^Tg(CAG-A4GALT)* Fabry model mice.

Fabry disease is an X-linked hereditary disease caused by mutations in *GLA* (α-galactosidase A) gene that encodes the lysosomal enzyme, GLA (1), and it is characterized by the systemic accumulation of glycosphingolipids, especially globotriaosylceramide (Gb3), in the lysosomes of various cell types (2). Approximately 750 *GLA* mutations have been identified to date (3). Fabry disease is categorized as classic or late onset, according to the presence or absence of early classic manifestations—acroparesthesia, clustered angiokeratoma, cornea verticillata, hypoanhidrosis, *etc*.—and the type of *GLA* mutation. Nephropathy is a main feature of both disease subtypes (4). The distribution of GLA in the normal human kidney varies according to cell type (5). Although it is absent in glomeruli and endothelial cells, it is highly expressed in all tubular segments and interstitial cells. Conspicuous podocyte vacuolization is highly pathognomonic, and podocyte injury is thought to play a critical role in the development and progression of Fabry nephropathy (6, 7). Progressive Gb3 deposition in the kidney results in proteinuria and the gradual deterioration of renal function and the development of azotemia (8), which affects all tubules, but especially the distal tubules for reasons that are unclear (9–11). No previous studies have used segment-specific Abs to clarify whether the affected segment is the thick ascending limb (TAL) or distal convoluted tubule (DCT) (9–11). Both glomerular and tubular injury occur in Fabry disease, and the effect of the latter alone on renal dysfunction is unclear.

Polyuria in Fabry disease was first reported in 1958 (12) and is prominent early in the disease course (6, 9, 12–16), although the affected tubules and underlying mechanism remain unknown (6, 13, 15, 16). *Gla* knockout (*Gla^tm^*) mice do not develop Fabry disease as a result of a lesser accumulation of Gb3 than that observed in humans (17). To increase Gb3 levels, we previously crossbred asymptomatic *Gla^tm^* mice (18, 19) with transgenic mice that expressed human Gb3 synthase (A4GALT) to generate the *Gla^tm^Tg(CAG-A4GALT)* symptomatic Fabry model mice. Additional analyses revealed that these mice exhibit polyuria and renal dysfunction without remarkable glomerular damage and provided the first clear evidence that Gb3 accumulation is the primary cause of Fabry disease (20). This mouse line is suitable for studying tubular injury that is directly caused by Gb3 accumulation. Autopsy specimens demonstrated Gb3 accumulation in the medullary TALs (mTALs) (21), which suggests that these are the most severely affected tubules. TAL plays critical roles in salt (Na^+^, K^+^, and Cl^−^) reabsorption, blood pressure (BP) control, urine concentration, and divalent cation (Ca^2+^ and Mg^2+^) homeostasis (22). We carried out the present study to clarify the mechanistic basis for polyuria and renal dysfunction in *Gla^tm^Tg(CAG-A4GALT)* mice and to examine TAL morphology in patients with Fabry nephropathy.

## MATERIALS AND METHODS

### Animal studies

#### Animals

The C57BL/6J-*Gla^tm1kul^Tg(CAG-A4GALT)* mouse line (20) was generated by crossbreeding C57BL/6J-*Tg(CAG-A4GALT*) mice that harbored the *A4GALT* transgene in a single allele with homozygous *Gla* knockout C57BL/6J;129S4-*Gla^tm1kul^* mice (18). The following nomenclature is used hereafter to describe mouse models: *Tg(A4GALT)* for C57BL/6J-*Tg(CAG-A4GALT)*, *Gla^tm^* for C57BL/6J;129S4-*Gla^tm1kul^*, and *Gla^tm^Tg(A4GALT)* for C57BL/6J-*Gla^tm1kul^Tg(CAG-A4GALT)*. Wild-type (WT) mice (C57BL/6J) were purchased from Charles River Laboratories (Yokohama, Japan). All mice were housed under standard laboratory conditions of 24 ± 2°C and 50–60% humidity on a 12-h light/dark cycle with free access to tap water and commercial standard rodent chow that contained 1.20% calcium, 1.08% phosphate, and 240 IU/100 g vitamin D_3_ (CE-2; Clea Japan, Tokyo, Japan). Mouse lines were genotyped by PCR amplification of *A4GALT* as described in Taguchi *et al*. (20). To eliminate the possibility that the phenotype could be affected by pregnancy, female mice were excluded from experiments. All animal protocols were reviewed by the institutional animal care and use committee and were approved by the Presidents of Niigata University (H21 Niigata University Research 69, H25-111 Niigata University Research 255-1, H27-182 Niigata University Research 323-6) and Oita University (P006002).

#### Blood analysis

Na, K, Cl, glucose, blood urea nitrogen, and creatinine (Cr) levels in whole blood were determined by using an i-Stat analyzer (Abbott, Tokyo, Japan). Total protein, albumin, uric acid, Ca, and Mg levels were measured by the Oriental Yeast Co. (Nagahama, Japan). Plasma osmolality was calculated by using the following formula: osmolarity = 1.86 (Na + K) + glucose + urea + 10 [with Na, K, glucose (mM)] (23).

#### Urine analysis

Mice were maintained in metabolic cages for 24 h of urine collection. Urine Na, K, Cl, Ca, Mg, urea nitrogen, and Cr were analyzed by the Oriental Yeast Company. Urine osmolality was measured and the fractional excretion (FE) of a solute X (FE_x_) was calculated by using the following formula: FE_x_ = ([X]_24 h urine_ × 24 h urine volume)/([X] _plasma_ × Cr clearance) × 100 (24). The level of 8-hydroxyl-2′-deoxyguanosine (8-OHdG) was measured by using the new 8-OHdG Check Eliza Kit (JaICA, Fukuroi, Japan).

#### Mouse kidney pathology

Kidneys were cut transversely and fixed in 10% neutral-buffered formalin, embedded in paraffin, and treated with periodic acid–Schiff (PAS) and elastica Masson trichrome stains. Deparaffinized sections were prepared by routine procedure. Immunohistochemical staining of formalin-fixed, paraffin-embedded kidneys was performed after antigen retrieval in Target Retrieval Solution (Dako, Glostrup, Denmark). Abs against the following proteins were used: uromodulin (UMOD; AbD Serotec, Raleigh, NC, USA), Na^+^-K^+^-2Cl^−^ cotransporter (NKCC2; StressMarq Biosciences, Victoria, BC, Canada), Na^+^-K^+^-ATPase (Abcam, Cambridge, United Kingdom), F4/80 (AbD Serotec), Na^+^-Cl^−^ cotransporter (NCC; EMD Millipore, Billerica, MA, USA), aquaporin-2 (AQP2; Abcam), and malondialdehyde (JaICA). All slides were counterstained with hematoxylin. We performed Gb3 staining with Shiga toxin 1 B subunit and transmission electron microscopy as described in Taguchi *et al*. (20). Toluidine blue staining was performed with 0.05% toluidine blue solution. We used an all-in-one fluorescence microscope (BZ-X700; Keyence, Osaka, Japan) to document staining. Information on Abs used in this study is provided in Supplemental Table 1.

#### Real-time RT-PCR analysis

Real-time RT-PCR was performed by using total RNA from the whole kidney along with appropriate primer sets and the One-Step SYBR PrimeScript Plus RT-PCR Kit (Takara Bio, Kusatsu, Japan) on a Thermal Cycler Dice Real Time System II (Takara Bio). Forward and reverse primer sequences used are as follows: glyceraldehyde 3-phosphate dehydrogenase (*Gapdh*): 5′-TGTGTCCGTCGTGGATCTGA-3′ and 5′-TTGCTGTTGAAGTCGCAGGAG-3′; *Umod*: 5′-GGTCCCTGTGGGACAGTATTGAG-3′ and 5′-GATGATCGCATTTGCCAGGTAG-3′; solute carrier family 12 member (*Slc12a*) *1*: 5′-GATGCAGAACTGGAAGCAGTCAA-3′ and 5′-GCCATGTACAACAAATCCGAAATAG-3′; transforming growth factor β (*Tgfb*) *1*: 5′-GTGTGGAGCAACATGTGGAACTCTA-3′ and 5′-CGCTGAATCGAAAGCCCTGTA-3′; collagen type I α 1 chain (*Col1a1*): 5′-GACATGTTCAGCTTTGTGGACCTC-3′ and 5′-GGGACCCTTAGGCCATTGTGTA-3′; collagen type III α 1 chain (*Col3a1*): 5′-CAGGCCAGTGGCAATGTAAAGA-3′ and 5′-CTCATTGCCTTGCGTGTTTGATA-3′; fibronectin (*Fn*) *1*: 5′-GCTTTGGCAGTGGTCATTTCAG-3′ and 5′-ATTCCCGAGGCATGTGCAG-3′; C-C motif chemokine ligand (*Ccl*) *2*: 5′-AGCAGCAGGTGTCCCAAAGA-3′ and 5′-GTGCTGAAGACCTTAGGGCAGA-3′; chemokine (C-X3-C motif) ligand (*Cx3cl*) *1*: 5′-ATCCGCTATCAGCTAAACCAGGAG-3′ and 5′-TCCCAGGTGTCACATTGTCCA-3′; adhesion G protein-coupled receptor E (*Adgre*) *1*: 5′-GAGATTGTGGAAGCATCCGAGAC-3′ and 5′-GACTGTACCCACATGGCTGATGA-3′; hepatocyte growth factor (*Hgf*): 5′-TCCATGTGGGACAAGAATATGGAG and 5′-CATCATCAGGATTCCGGCAGTA-3′; *Slc12a3*: 5′-TCGGCAGGTGAGACTGAGTGA-3′ and 5′-GTCTCCAGCCAGGCCATGTA-3′; and *Aqp2*: 5′-AGCTGGTGCTGTGCATCTTTG-3′ and 5′-ATGGAGCAGCCGGTGAAATA-3′. mRNA expression levels were calculated as the inverted cycle threshold relative to the level of *Gapdh*.

#### Western blot analysis

Whole-kidney homogenates were adjusted to the same protein concentrations as determined by the DC Protein Assay (Bio-Rad, Hercules, CA, USA). Abs against the following proteins were used: UMOD (AbD Serotec), NKCC2 (Alpha Diagnostic International, San Antonio, TX, USA), Na^+^-K^+^-ATPase (Abcam), NCC (EMD Millipore), AQP2 (Abcam), arginine vasopressin (AVP) receptor 2 (Alomone Labs, Jerusalem, Israel), and GAPDH (MilliporeSigma, St. Louis, MO, USA). Abs were diluted with Western Blot Immuno Booster (Takara Bio). Immunoreaction was detected with SuperSignal West Pico Chemiluminescent Substrate (Thermo Fisher Scientific, Waltham, MA, USA), and protein bands were scanned with an ImageQuant LAS 4000 Mini (GE Healthcare, Piscataway, NJ, USA) and quantified by using ImageQuant TL v.8.1 software (GE Healthcare). Information on the Abs used in this study is provided in Supplemental Table 1.

#### Other assays

Plasma AVP concentration was measured by using the Arg^8^-Vasopressin Chemiluminescent Immunoassay Kit (Arbor Assays, Ann Arbor, MI, USA). ATP concentration in kidneys was measured by using an ATP Assay Kit (Toyo Ink, Tokyo, Japan). BP—an average of 3 consecutive measurements—was measured with the tail-cuff method using a programmable sphygmomanometer (Softron, Tokyo, Japan). To eliminate the effect of environmental changes on BP, mouse handlers measured BP in a quiet rearing room. Vendor-derived WT mice were allowed to acclimate to the rearing environment for at least 1 wk upon arrival before measurements were taken. Each mouse was gently immobilized in a mouse holder and maintained at 39°C in the warmer >5 min before measurements were taken to allow the mouse to acclimate to the holder and to increase blood flow to the tail artery. As most mice adapted to measurements, there was no prior training. When a mouse did not remain quiet, BP was measured the next day or later.

#### Statistical analysis and graph preparation

At least 5 mice/group were used for all studies, except for histologic analyses. Two-tailed significance values are reported. We used the Shapiro–Wilk test to test for normal distribution. Normally distributed data were evaluated for variance with the *F* test. Statistical analyses—Student’s *t* test, Welch’s *t* test, and Wilcoxon rank-sum test—were performed using JMP v.12 (SAS Institute, Cary, NC, USA). Values of *P* < 0.05 were considered statistically significant. SigmaPlot v.12.5 (Systat Software, San Jose, CA, USA) was used to draw graphs.

### Human studies

#### Informed consent and ethics

The study protocol was approved by the Ethics Committee of the Niigata University School of Medicine (H27-2137 and H28-2414) and the collaborating hospitals in accordance with the Declaration of Helsinki, and written informed consent was obtained from all patients.

#### Human kidney pathology

PAS and elastica Masson trichrome staining were performed as described above. Immunohistochemical staining was performed with the HRP-DAB System Cell and Tissue Staining Kit (R&D Systems, Minneapolis, MN, USA) and the above-mentioned primary Abs after antigen retrieval in Target Retrieval Solution. Toluidine blue staining and transmission electron microscopy were performed as described above with minor differences in reagents and devices. Information on Abs used is provided in Supplemental Table 2.

## RESULTS

### Gb3 accumulates in various renal tubules

**Figure 1:**
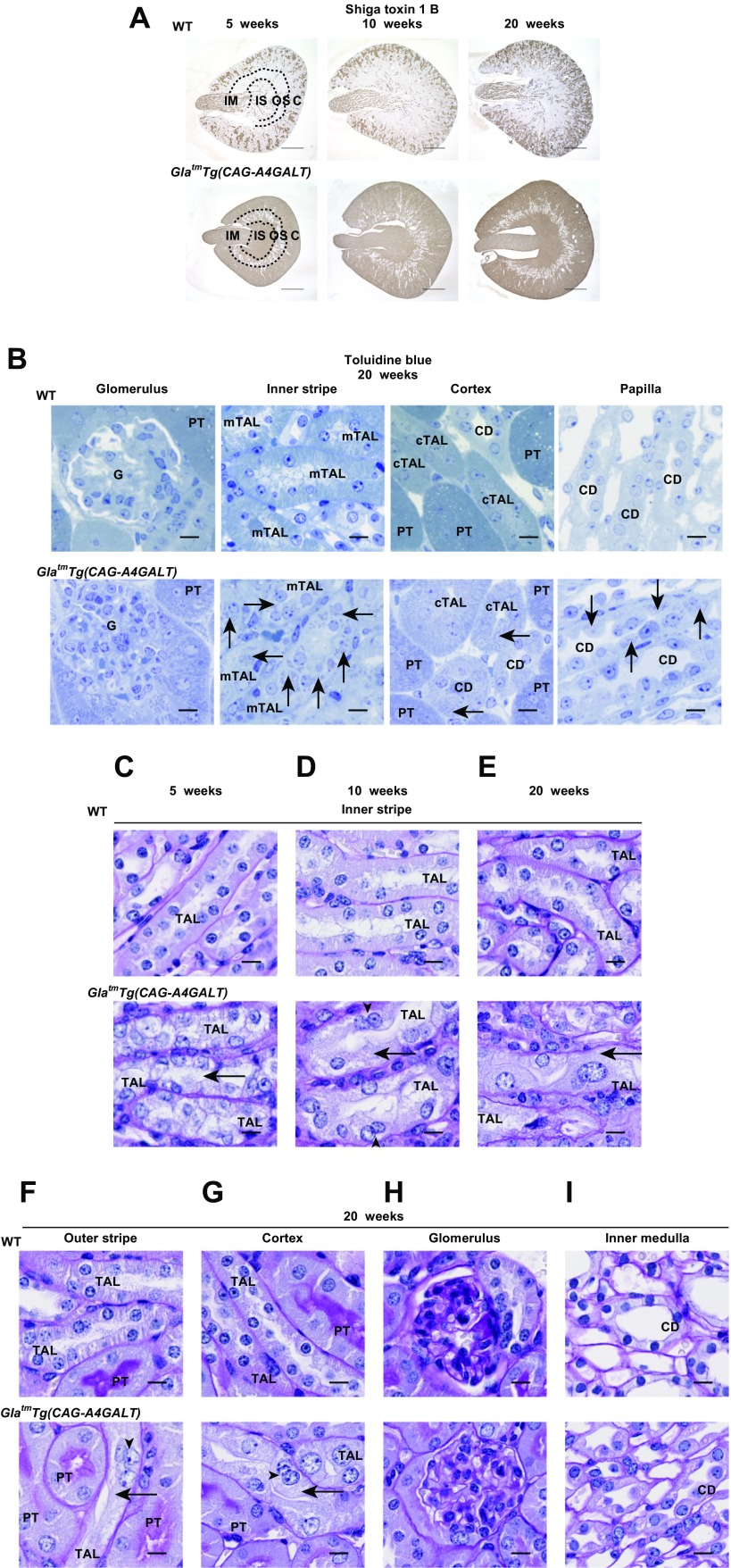
*Gla^tm^Tg(CAG-A4GALT)* mice exhibit mTAL impairment. *A*) Representative micrographs of the transverse plane of kidneys from *Gla^tm^Tg(CAG-A4GALT)* and WT mice stained for Gb3 with Shiga toxin 1 B subunit (*n* = 3/group). Scale bars, 1 mm. *B*) Representative micrographs of kidneys of *Gla^tm^Tg(CAG-A4GALT)* and WT mice stained for Gb3 accumulation in lysosomes with toluidine blue (*n* = 3/group). Arrows indicate positively stained dots. Scale bars, 10 μm. *C*–*E*) Representative micrographs of PAS staining of TALs in the inner stripe of *Gla^tm^Tg(CAG-A4GALT)* and WT mice (*n* = 3/group). *F*–*I*) Micrographs of PAS staining of TALs in the outer stripe, TALs in the cortex, glomerulus, and CD in the inner medulla of *Gla^tm^Tg(CAG-A4GALT)* and WT mice. Arrows indicate a vacuolated TAL cell, and arrowheads indicate a binuclear TAL cell. Scale bars, 10 μm. C, cortex; cTAL, cortical TAL; G, glomerulus; IM, inner medulla; IS, inner stripe of the outer medulla; OS, outer stripe of the outer medulla; PT, proximal tubule.

To examine the global effect of Gb3 overload on kidneys, kidney sections were stained for Gb3 by using Shiga toxin 1 B subunit. In WT mice, the collecting duct (CD) area was positive for Gb3 (**Fig. 1*A***), as previously reported (25). In *Gla^tm^Tg(CAG-A4GALT)* mice, staining progressively spread to and was enhanced in all tubules, except for many in the outer stripe (OS) (Fig. 1*A*). Differences in Gb3 accumulation between strains were mostly detected in the outer and inner medulla, which indicates that these areas were the main affected sites. Gb3 that were stained with toluidine blue in lysosomes (19) appeared as dots, especially in TALs and CDs in the outer and inner medulla (Fig. 1*B*). There was no staining in glomeruli. These data are consistent with the finding that Gb3 is not only present in lysosomes, but is also widely distributed in extralysosomal structures, even in the absence of lysosomal inclusions as observed in patients with Fabry disease (26).

### Vacuolization predominantly appears in mTALs

Vacuolization of TAL in *Gla^tm^Tg(CAG-A4GALT)* mice was predominant in the inner stripe (IS), followed by the OS, and was sparse in the cortex of 5-wk-old mice with later progression (Fig. 1*C*–*G*). The nucleus and cytoplasm of TAL cells swelled, whereas the lumen narrowed with development (Fig. 1*C*–*E*). Cellular infiltration was prominent around impaired TALs (Fig. 1*C*–*E*). Some TAL cells were binuclear, which indicates regeneration (Fig. 1*C*–*G*). Vacuolization was absent from proximal tubules and glomeruli (Fig. 1*F*–*H*), and some CD cells were swollen (Fig. 1*I*). These observations suggest that, apart from the Gb3 overload, mTAL cells also possess certain other characteristics that contribute to the severe effect on mTALs.

### TAL dysfunction induces water- and salt-loss phenotypes

**Figure 2:**
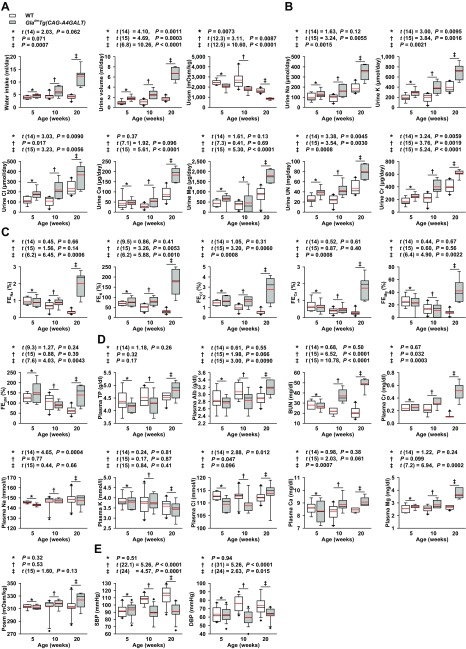
*Gla^tm^Tg(CAG-A4GALT)* mice exhibit a water- and salt-loss phenotype. *A*) Water intake, urine volume, and urine osmolality (Uosm). *B*) Daily urinary excretion of solutes. *C*) FE of solutes. *D*) Blood chemistry. Total protein (TP), albumin (Alb), blood urea nitrogen (BUN), Cr, Na, K, Cl, Ca, Mg, and plasma osmolality (Posm). *Gla^tm^Tg(CAG-A4GALT)*: 5 (*n* = 8), 10 (*n* = 7), and 20 wk old (*n* = 7); WT mice: 5 (*n* = 8), 10 (*n* = 10), and 20 wk old (*n* = 10). *E*) Systolic (S) BP and diastolic (*D*) BP. *Gla^tm^Tg(CAG-A4GALT)* mice: 5 (*n* = 19), 10 (*n* = 16), and 20 wk old (*n* = 12); WT mice: 5 (*n* = 20), 10 (*n* = 17), and 20 wk old (*n* = 14). In box-and-whisker plots, center lines represent the median, limits represent quartiles, whiskers represent the 10th and 90th percentiles, black diamonds represent minima or maxima outside the reach of whiskers, and red lines represent the mean. Differences between groups were evaluated by using Student’s *t* test; data are shown as *t* (integral degree of freedom) = *t*, *P*. For Welch’s *t* test, data are shown as *t* (mixed decimal degree of freedom) = *t*, *P*. For the Wilcoxon rank-sum test, data are shown with a *P* value only.

*Gla^tm^Tg(CAG-A4GALT)* mice were characterized by progressive polyuria, polydipsia, and decreased urine osmolality (**Fig. 2*A***), which indicates an inability to concentrate urine. Daily urinary excretion of solutes in *Gla^tm^Tg(CAG-A4GALT)* mice increased progressively (Fig. 2*B*). TAL reabsorbs Na^+^, K^+^, and Cl^−^ *via* transepithelial transport and Ca^2+^ and Mg^2+^ *via* paracellular transport (22). FE is a parameter that reflects the tubular reabsorption of solutes; the FE of a solute X (FE_x_) expresses the amount of solute X that is excreted in the urine as a percentage of the amount of filtered X. A decrease in tubular reabsorption of X leads to high FE_x_. *Gla^tm^Tg(CAG-A4GALT)* mice displayed a higher FE of solutes than did WT mice by age 20 wk (Fig. 2*C*), which resulted in progressive water- and salt-loss phenotypes; all findings related to electrolytes could be attributed to TAL dysfunction (27). Water and salt loss affected the blood chemistry values of *Gla^tm^Tg(CAG-A4GALT)* mice (Fig. 2*D*) and led to progressive renal dysfunction (Fig. 2*D*). As expected, these mice had a lower BP than did WT mice by age 10 wk (Fig. 2*E*).

### Decreased levels of UMOD, *Umod*, NKCC2, *Slc12a1*, and Na^+^-K^+^-ATPase in TALs

**Figure 3:**
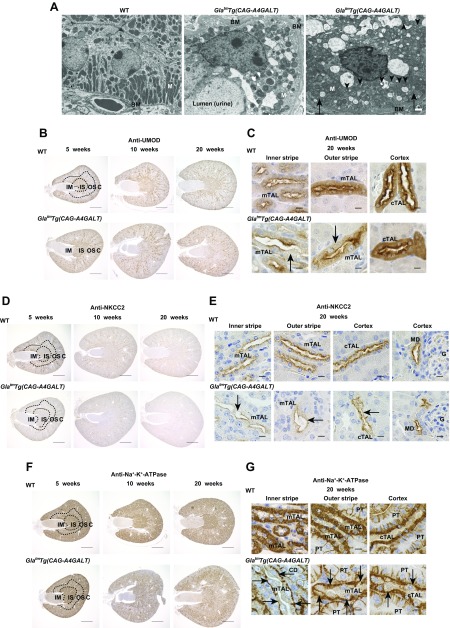
*Gla^tm^Tg(CAG-A4GALT)* mice demonstrate reduced expression of ion transport–related molecules in the TAL. *A*) Representative transmission electron micrographs of the mTAL in 20-wk-old *Gla^tm^Tg(CAG-A4GALT)* and WT mice (*n* = 2/group). Arrows indicate basolateral infolding, and arrowhead indicates a lamellar body. Scale bars, 1 μm. *B*) Representative images of UMOD immunoreactivity in kidneys of *Gla^tm^Tg(CAG-A4GALT)* and WT mice (*n* = 3/group). Scale bars, 1 mm. *C*) Micrographs of UMOD expression in TAL of *Gla^tm^Tg(CAG-A4GALT)* and WT mice. Arrows indicate a vacuolated TAL cell. Scale bars, 10 μm. *D*) Representative images of NKCC2 immunoreactivity in kidneys of *Gla^tm^Tg(CAG-A4GALT)* and WT mice (*n* = 3/group). Scale bars, 1 mm. *E*) Micrographs of NKCC2 expression in TAL of *Gla^tm^Tg(CAG-A4GALT)* and WT mice. Arrows indicate TAL cells with weak NKCC2 staining. Scale bars, 10 μm. *F*) Representative images of Na^+^-K^+^-ATPase immunoreactivity in kidneys of *Gla^tm^Tg(CAG-A4GALT)* and WT mice (*n* = 3/group). Scale bars, 1 mm. *G*) Micrographs of Na^+^-K^+^-ATPase expression in TAL of *Gla^tm^Tg(CAG-A4GALT)* and WT mice. Arrows indicate TAL cells with weak Na^+^-K^+^-ATPase staining. Scale bars, 10 μm. BM, basement membrane; G, glomerulus; M, mitochondria; MD, macula densa.

TAL cells in WT mice demonstrated extensive infolding and numerous elongated mitochondria (**Fig. 3*A***) (28). In contrast, we observed lamellar bodies, round mitochondria, and disorganized, flattened infoldings in mTAL cells of *Gla^tm^Tg(CAG-A4GALT)* mice (Fig. 3*A*).

The TAL-specific protein, UMOD, facilitates membrane trafficking and NKCC2 protein function (29). In WT mice, UMOD was densely distributed in the IS (Fig. 3*B*), but was weakly expressed on the apical and basolateral membranes in the IS (Fig. 3*C*). The protein was sparsely distributed (Fig. 3*B*) but demonstrated a stronger signal in the OS and cortex (Fig. 3*C*). In *Gla^tm^Tg(CAG-A4GALT)* mice, UMOD expression was reduced in the outer medulla (IS > OS) by age 10 wk, with a progressive decrease in levels thereafter (Fig. 3*B, C*).

NKCC2 is important for Na^+^-Cl^−^ reabsorption, which is required to maintain a high interstitial osmolality for countercurrent multiplication and water reabsorption by CDs as well as for divalent cation transport in TALs (27). In WT mice, NKCC2 was broadly distributed (Fig. 3*D*) and expressed in the apical domain of the TAL and macula densa (Fig. 3*E*); however, in *Gla^tm^Tg(CAG-A4GALT)* mice, levels were lower in the outer medulla (IS > OS) by age 10 wk and progressively decreased (Fig. 3*D*). NKCC2 expression persisted in the macula densa (Fig. 3*E*), which suggests that these cells retained normal function.

**Figure 4:**
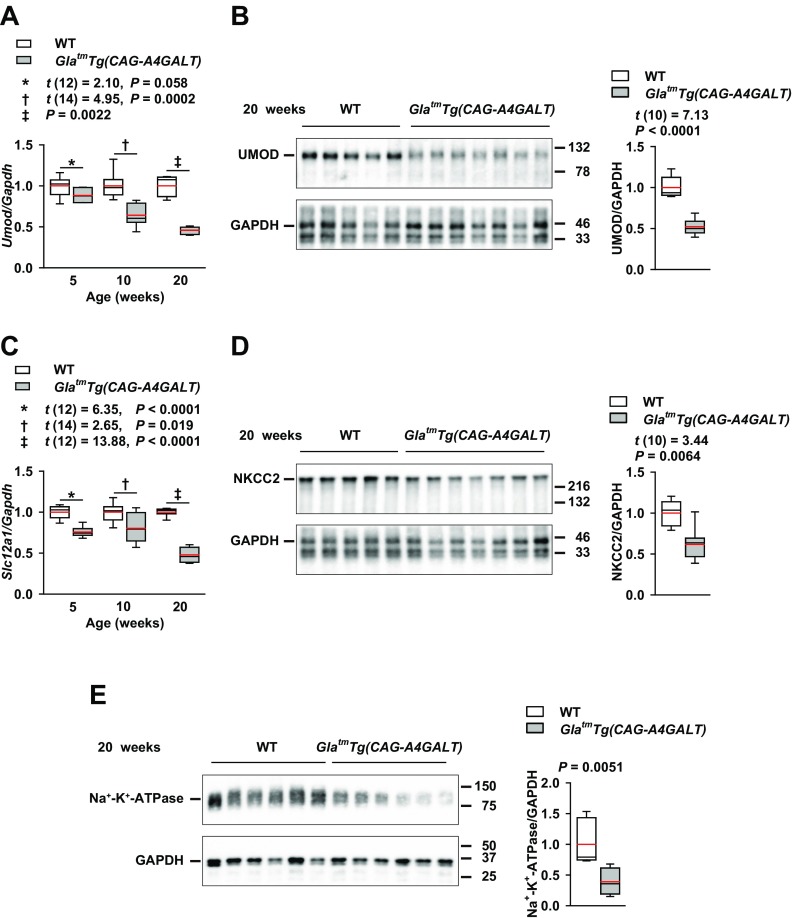
*Gla^tm^Tg(CAG-A4GALT)* mice demonstrate reduced expression of *Umod* (UMOD), *Slc12a1* (NKCC2), and Na^+^-K^+^-ATPase in the whole kidney. *A*) Real-time RT-PCR analysis of *Umod* mRNA levels in *Gla^tm^Tg(CAG-A4GALT)* and WT mice. *Gla^tm^Tg(CAG-A4GALT)* mice: 5 (*n* = 7), 10 (*n* = 8), and 20 wk old (*n* = 7); WT mice: 5 (*n* = 7), 10 (*n* = 8), and 20 wk old (*n* = 7). *B*) Representative (of 2 experiments) Western blot analysis of UMOD expression in *Gla^tm^Tg(CAG-A4GALT)* (*n* = 7) and WT mice (*n* = 5). *C*) Real-time RT-PCR analyses for *Slc12a1* expression levels in the same mice as in panel *A*. *D*) Representative (of 2 experiments) Western blot analysis of NKCC2 expression in the same mice as in panel *B*. *E*) Representative (of 2 experiments) Western blot analysis of Na^+^-K^+^-ATPase expression in *Gla^tm^Tg(CAG-A4GALT)* (*n* = 6) and WT mice (*n* = 6). In box-and-whisker plots, center lines represent the median, limits represent quartiles, whiskers represent the 10th and 90th percentiles, and red lines represent the mean. Differences between groups were evaluated by using Student’s *t* test; data are shown as *t* (integral degree of freedom) = *t*, *P*. For Welch’s *t* test, data are shown as *t* (mixed decimal degree of freedom) = *t*, *P*. For the Wilcoxon rank-sum test, data are shown with a *P* value only.

Na^+^-K^+^-ATPase, which mediates Na^+^ reabsorption, is highly expressed in the basolateral membrane of the highly Na^+^-reabsorbing nephrons, DCTs, and TALs (30). In WT mice, Na^+^-K^+^-ATPase distribution was dense in the IS and sparser in the OS and cortex (Fig. 3*F*), whereas, in TALs, there was high expression that clearly delineated cell morphology (Fig. 3*G*). In *Gla^tm^Tg(CAG-A4GALT)* mice, staining intensity was reduced in the IS, and the area with a lower signal progressively expanded (Fig. 3*F, G*), which indicates that impaired Na^+^ transport systems contribute to TAL dysfunction. This was supported by the observation that *Umod* mRNA and UMOD protein, *Slc12a1* mRNA, and NKCC2 protein levels (**Fig. 4*A*–*D***), along with Na^+^-K^+^-ATPase expression (Fig. 4*E*), were significantly lower in *Gla^tm^Tg(CAG-A4GALT)* mice compared with WT mice.

### Fibrosis and inflammation are detected surrounding impaired TALs

**Figure 5:**
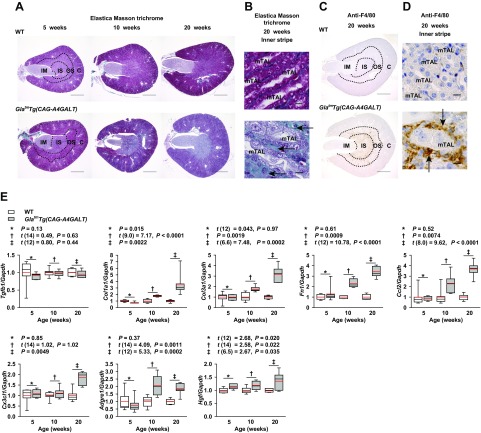
*Gla^tm^Tg(CAG-A4GALT)* mice demonstrate fibrosis and inflammation. *A*) Representative micrographs of elastica Masson trichrome staining in kidneys of *Gla^tm^Tg(CAG-A4GALT)* and WT mice (*n* = 3/group). Scale bars, 1 mm. *B*) Micrographs of elastica Masson trichrome staining of the inner stripe of *Gla^tm^Tg(CAG-A4GALT)* and WT mice. Arrows indicate interstitial fibrosis. Scale bars, 10 μm. *C*) Representative micrographs of F4/80-positive macrophage infiltration in *Gla^tm^Tg(CAG-A4GALT)* and WT mice (*n* = 3/group). Scale bars, 1 mm. *D*) Micrographs of F4/80-positive macrophages in the inner stripe of *Gla^tm^Tg(CAG-A4GALT)* and WT mice. Arrows indicate F4/80-positive macrophages. Scale bars, 10 μm. *E*) Real-time RT-PCR analysis of the expression of genes related to fibrosis [transforming growth factor β (*Tgfb*)*1*, collagen type I α 1 chain (*Col1a1*), collagen type III α 1 chain (*Col3a1*), and fibronectin (*Fn*) *1*], inflammation [C-C motif chemokine ligand (*Ccl*) *2*, chemokine (*C-X3-C motif*) ligand (*Cx3cl*) *1*, and adhesion G protein-coupled receptor E (*Adgre*) *1*], and regeneration [hepatocyte growth factor (*Hgf*)] in the whole kidney of the same mice as in Fig. 4*A*. In box-and-whisker plots (*E*), center lines represent the median, limits represent quartiles, whiskers represent the 10th and 90th percentiles, and red lines represent the mean. Differences between groups were evaluated by using Student’s *t* test; data are shown as *t* (integral degree of freedom) = *t*, *P*. For Welch’s *t* test, data are shown as *t* (mixed decimal degree of freedom) = *t*, *P*. For the Wilcoxon rank-sum test, data are shown with a *P* value only.

Fibrosis is an early event in the course of Fabry nephropathy (31). In the tubular cytoplasm of *Gla^tm^Tg(CAG-A4GALT)* mice, elastica Masson trichrome staining became progressively weaker (**Fig. 5*A, B***), which is similar to the gradual accumulation of Gb3. Focal fibrosis was detected around TALs in the outer medulla (IS > OS) of *Gla^tm^Tg(CAG-A4GALT)* mice by age 10 wk and progressed with age (Fig. 5*A, B*). Macrophage-specific antigen F4/80–positive cells infiltrated into and around TALs, then spread throughout the tissue in *Gla^tm^Tg(CAG-A4GALT)* mice (Fig. 5*C, D*). Transcript levels of the major fibrosis, inflammation, and regeneration markers were up-regulated (Fig. 5*E*).

### Downstream tubular compensatory response to salt- and water-loss phenotypes induced by TAL dysfunction

**Figure 6:**
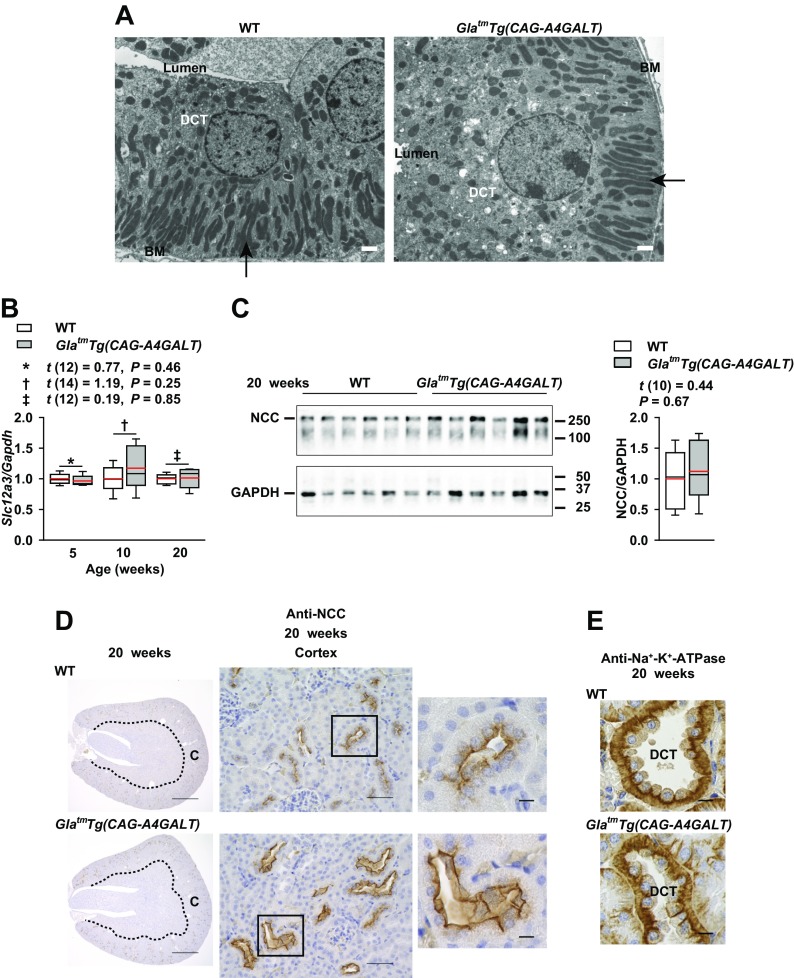
DCT response to TAL dysfunction. *A*) Transmission electron micrographs of a DCT in 20-wk-old *Gla^tm^Tg(CAG-A4GALT)* and WT mice. Arrows indicate elongated mitochondria and an extensive basolateral membrane (infolding). Scale bars, 1 μm. *B*) Real-time RT-PCR analysis of *Slc12a3* (NCC) in the same mice as in Fig. 4*A*. *C*) Western blot analysis of NCC expression in the same mice as in Fig. 4*B*. *D*) Representative images of NCC immunoreactivity in *Gla^tm^Tg(CAG-A4GALT)* and WT mice (*n* = 3/group). Scale bars, 1 mm (left), 50 μm (middle), and 10 μm (right). *E*) Representative images of Na^+^-K^+^-ATPase immunoreactivity in a DCT of *Gla^tm^Tg(CAG-A4GALT)* and WT mice (*n* = 3/group). Scale bars, 10 μm. In box-and-whisker plots (*B*, *C*), center lines represent the median, limits represent quartiles, whiskers represent the 10th and 90th percentiles, and red lines represent the mean. Differences between groups were evaluated with the Student’s *t* test; data are shown as *t* (integral degree of freedom) = *t*, *P*. For Welch’s *t* test, data are shown as *t* (mixed decimal degree of freedom) = *t* , *P*. For the Wilcoxon rank-sum test, data are shown with a *P* value only.

Enhanced Na^+^ delivery to the DCT is thought to stimulate DCT hypertrophy as a compensatory response, which manifests as increases in mitochondrial size and infolding (32, 33). DCT cells had a broad apical cytoplasmic region with vesicles, but there were no increases in mitochondrial size and infolding in *Gla^tm^Tg(CAG-A4GALT)* mice (**Fig. 6*A***). Thus, these mice demonstrated hypertrophy of DCT cells, but lacked some of its features (32, 33). NCC (*Slc12a3*) is a DCT-specific Na^+^-Cl^−^ cotransporter that plays an important in fine-tuning Na^+^ excretion (34, 35). *Slc12a3* mRNA and total NCC protein levels did not differ significantly between *Gla^tm^Tg(CAG-A4GALT)* and WT mice (Fig. 6*B, C*). NCC staining of the DCT revealed a wider lumen, increased cell size, and decreased cell density, which indicates hypertrophy (Fig. 6*D*), and not hyperplasia (36), of DCT cells. NCC-positive lumen surface area was greater and more prominent in *Gla^tm^Tg(CAG-A4GALT)* mice (Fig. 6*D*), which suggests a compensatory response. DCT cells are enriched in mitochondria and have the highest density of Na^+^-K^+^-ATPase in the kidney (30); Na^+^-K^+^-ATPase signals in DCT cells were similar between *Gla^tm^Tg(CAG-A4GALT)* and WT mice (Fig. 6*E*).

**Figure 7:**
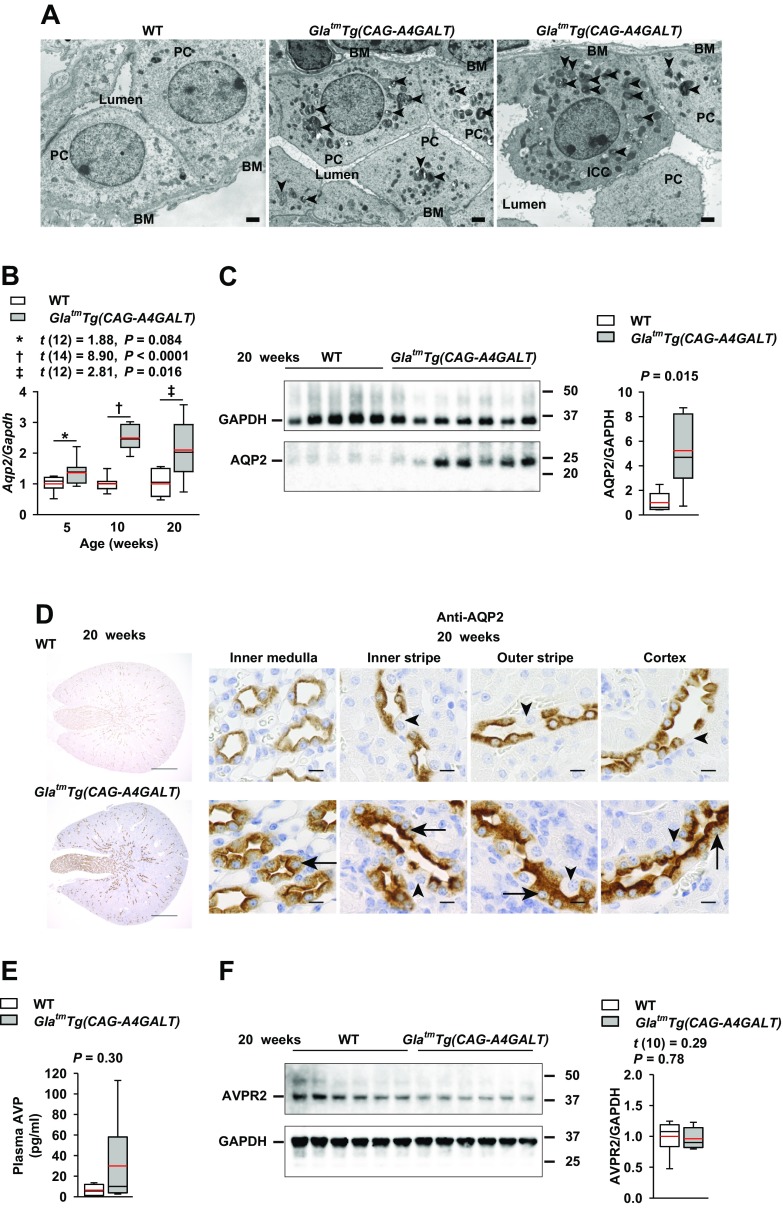
CD response to TAL dysfunction. *A*) Representative transmission electron micrographs of a CD in the inner medulla in 20-wk-old *Gla^tm^Tg(CAG-A4GALT)* and WT mice (*n* = 3/group). Arrowheads indicate a lamellar body. Scale bars, 1 μm. *B*) Real-time RT-PCR analysis of *Aqp2* levels in the same mice as in Fig. 4*A*. *C*) Representative (of 2 experiments) Western blot analysis of AQP2 in the same mice as in Fig. 4*B*. *D*) Representative images of a PC-specific transporter (AQP2) in a CD of *Gla^tm^Tg(CAG-A4GALT)* and WT mice (*n* = 3/group). Arrows indicate a swollen PC. Arrowheads indicate an ICC. Scale bars, 10 μm. *E*) Plasma AVP levels in 20-wk-old *Gla^tm^Tg(CAG-A4GALT)* and WT mice (*n* = 6/group). *F*) Representative (of 2 experiments) Western blot analysis of AVP receptor 2 (AVPR2) expression in *Gla^tm^Tg(CAG-A4GALT)* and WT mice (*n* = 6/group). In box-and-whisker plots (*B*, *C*, *E*, *F*), center lines represent the median, limits represent quartiles, whiskers represent the 10th and 90th percentiles, and red lines represent the mean. Differences between groups were evaluated by using Student’s *t* test; data are shown as *t* (integral degree of freedom) = *t*, *P*. For Welch’s *t* test, data are shown as *t* (mixed decimal degree of freedom) = *t*, *P*. For the Wilcoxon rank-sum test, data are shown with a *P* value only. ICC, intercalated cell (mitochondria-rich, dark cell); PC, principal cell (light cell).

Both principal and mitochondria-rich intercalated cells in *Gla^tm^Tg(CAG-A4GALT)* mice displayed accumulation of lamellar bodies, but normal nuclei (**Fig. 7*A***). AQP2 is a water channel that is responsible for water reabsorption in CDs (37). *Aqp2* mRNA and AQP2 protein were up-regulated in *Gla^tm^Tg(CAG-A4GALT)* mice (Fig. 7*B, C*). Principal cells in the *Gla^tm^Tg(CAG-A4GALT)* medulla were swollen, but demonstrated normal AQP2 staining at the apical membrane and in the cytoplasm throughout the kidney (Fig. 7*D*). Although plasma AVP (Fig. 7*E*) levels in *Gla^tm^Tg(CAG-A4GALT)* and WT mice were not significantly different, they showed a higher trend in *Gla^tm^Tg(CAG-A4GALT)* mice. AVP receptor 2 (Fig. 7*F*) protein levels did not differ significantly between *Gla^tm^Tg(CAG-A4GALT)* and WT mice.

### Oxidative stress and impaired energy metabolism

**Figure 8:**
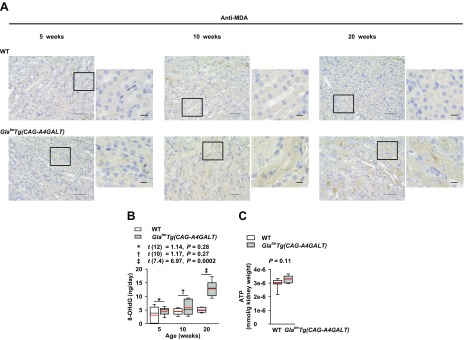
Oxidative stress in *Gla^tm^Tg(CAG-A4GALT)* mice. *A*) Representative images of malondialdehyde (MDA) immunoreactivity in *Gla^tm^Tg(CAG-A4GALT)* and WT mice (*n* = 3/group). Scale bars, 50 (left) and 10 μm (right). *B*) Time course of 24 h of urinary excretion of 8-OHdG in *Gla^tm^Tg(CAG-A4GALT)* and WT mice. *Gla^tm^Tg(CAG-A4GALT)* mice: 5 (*n* = 7), 10 (*n* = 6), and 20 wk old (*n* = 7); WT mice: 5 (*n* = 7), 10 (*n* = 6), and 20 wk old (*n* = 7). *C*) ATP content in the whole kidney of 20-wk-old *Gla^tm^Tg(CAG-A4GALT)* (*n* = 6) and WT (*n* = 9) mice. In box-and-whisker plots (*B*, *C*), center lines represent the median, limits represent quartiles, whiskers represent the 10th and 90th percentiles, and red lines represent the mean. Differences between groups were evaluated by using Student’s *t* test; data are shown as *t* (integral degree of freedom) = *t*, *P*. For Welch’s *t* test, data are shown as *t* (mixed decimal degree of freedom) = *t*, *P*. For the Wilcoxon rank-sum test, data are shown with a *P* value only.

Oxidative stress plays an important role in the pathophysiology of Fabry disease (38). Malondialdehyde, a lipid oxidative stress marker, was detected in mTAL of *Gla^tm^Tg(CAG-A4GALT)* mice by age 10 wk (**Fig. 8*A***). This coincided with changes in UMOD and NKCC2 expression (Fig. 3*B, D*) and renal injury (Fig. 2). Urinary excretion of 8-OHdG, a mitochondrial oxidative stress marker, progressively increased in *Gla^tm^Tg(CAG-A4GALT)* mice (Fig. 8*B*), which suggests that impaired mitochondria resulted in oxidative stress. Na^+^-K^+^-ATPase accounts for most of the energy consumption in the kidneys (39); we found that renal ATP content was similar between *Gla^tm^Tg(CAG-A4GALT)* and WT mice (Fig. 8*C*). The lower ATP production by impaired TAL mitochondria may have been offset by reduced ATP consumption that was a result of the down-regulation of Na^+^-K^+^-ATPase in TAL.

### TAL impairment in patients with Fabry disease

**Figure 9:**
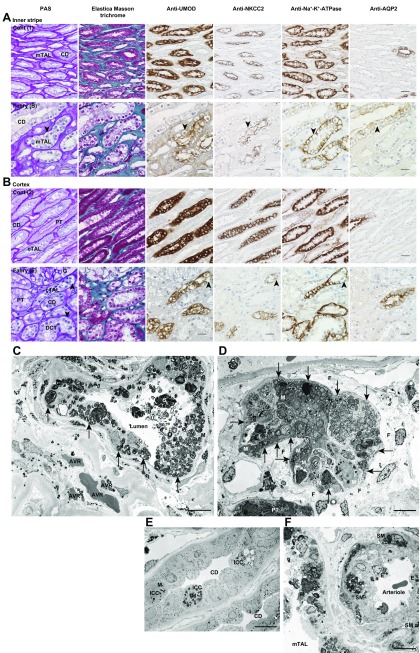
Micrographs of kidney biopsy serial sections (*A*, *B*) and transmission electron micrographs of kidney biopsy sections (*C*–*F*) from patients with Fabry nephropathy. PAS, elastica Masson trichrome, anti-UMOD Ab, anti-NKCC2 Ab, anti-Na^+^-K^+^-ATPase Ab, and anti-AQP2 Ab staining. *A*) Inner stripe: upper row, control [Cont (1)] lower row, Fabry nephropathy [Fabry (B)]. *B*) Cortex: upper row, control [Cont (2)]; lower row, Fabry nephropathy [Fabry (E)]. Impaired cells displayed vacuolization by PAS staining and attenuated or negative immunoreactivity (arrowheads). Fabry nephropathy patients were naive to enzyme replacement therapy at the time of kidney biopsy. Profiles of Fabry nephropathy [Fabry (B) and (E)] and control [Cont (1) and (2)] patients are shown in Supplemental Tables 3 and 4, respectively. Scale bars, 20 μm. *C*) mTAL of patient A. *D*) Cortical TAL (cTAL) of patient I. *E*) CD of patient A. *F*) Arteriole of patient A. Arrows indicate basolateral infolding. Scale bars, 10 μm. AVR, ascending vasa recta; E, endothelial cell; F, fibrosis; G, glomerulus; ICC, intercalated cell; M, mitochondria; S, sloughed lamellar body; SM, smooth muscle cell.

**Figure 10:**
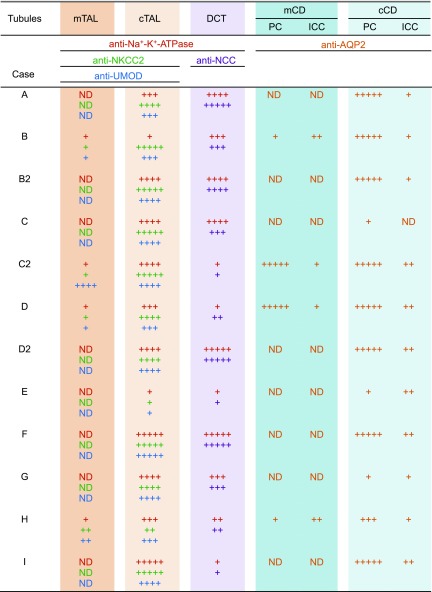
Attenuated immunoreactivity in renal tubules. Immunoreactivity was scored semiquantitatively according to the proportion of affected cells of the most impaired nephron in the sample as follows: +++++, normal staining; ++++, attenuated staining ≤1/4 of cells; +++, >1/4 and ≤1/2 of cells; ++, >1/2 and ≤3/4 of cells; and +, >3/4 of cells. Scattered intercalated cells (ICCs) were scored separately as follows: ++, normal staining; +, attenuated staining. Profiles of patients with Fabry nephropathy are presented in Supplemental Table 3. cCD, cortical CD; cTAL, cortical TAL; mCD, medullary CD; ND, not detectable; PC, principal cell.

We investigated TAL impairment in 9 patients with Fabry disease (Supplemental Table 3). Biopsied specimens that consisted of the cortex and medulla of 3 patients with non-Fabry chronic kidney disease served as control (Supplemental Table 4). In patients with Fabry disease (**Fig. 9*A, B***), PAS staining revealed podocyte vacuolization that appeared as a lacy expanded cytoplasm, as described in Colvin (40). TAL and DCT vacuoles were pale. Tubular cytoplasm demonstrated weak elastica Masson trichrome staining, and collagen was abundant around vacuolated tubules, which indicates fibrosis. Vacuolated TAL cells were weakly positive or negative for UMOD immunoreactivity, whereas NKCC2 expression was attenuated in the apical membrane and cytoplasm. Na^+^-K^+^-ATPase was down-regulated in the basolateral membrane. The degree to which UMOD, NKCC2, and Na^+^-K^+^-ATPase expression was decreased in impaired TAL cells varied. AQP2 was weakly detected in the apical membrane and cytoplasm of principal cells, and vacuolization was prominent in swollen AQP2-negative intercalated cells in CDs, as previously reported (10). Although disease severity varied among patients, semiquantification of tubular impairment indicated that mTALs were most severely impaired, followed by DCTs and cortical TALs (**Fig. 10**). A comparison of the first biopsy (B) and a second biopsy performed 15 yr later (B2) demonstrated that mTAL (B) was more severely impaired than cortical TAL (B2).

TAL cells contained lamellar bodies (Fig. 9*C, D*); infolding was disorganized and confined to the basal side, and mitochondria were reduced in number and were smaller than normal. In CDs, mitochondria-rich intercalated cells exhibited mitochondrial abnormalities and contained lamellar bodies (Fig. 9*E*), which were also present in endothelial and smooth muscle cells of the microvasculature (Fig. 9*C, F*).

## DISCUSSION

Using *Gla^tm^Tg(CAG-A4GALT)* Fabry model mice with polyuria, we have demonstrated that mTALs, especially in the IS, were the main tubules that were affected and that TAL dysfunction—an inability to concentrate urine—as a result of decreased expression of core molecules (Na^+^-K^+^-ATPase, UMOD, and NKCC2) induced water- and salt-loss phenotypes. Similar pathologic changes were observed in patients with Fabry disease. Given the critical roles played by TAL (22) and the resistance of distal tubules to enzyme replacement therapy (41), the identification of mTAL as the affected tubule in Fabry disease is significant.

mTALs may be targeted as a result of the characteristics of highly metabolic TAL cells (42), including their structure—extensive basolateral membrane and enrichment of mitochondria—and Na^+^ reabsorption function [Na^+^-K^+^-ATPase accounts for over 50% of energy consumed by the kidney (39)], and low medullary circulation (43, 44). As TALs of the IS are located outside the vascular bundles, their oxygen supply is limited, which makes them vulnerable to hypoxic injury (35). Gb3 accumulation in the microvasculature may affect luminal patency, inducing a proinflammatory and procoagulant response that leads to ischemic injury (40). The current study suggests that oxidative stress that results from mitochondrial impairment because of Gb3 accumulation and the limited blood supply in the IS (43) may synergistically affect TAL cells (45). Mitochondria-rich DCT cells (9–11) and intercalated cells (21) may be similarly affected.

TAL plays a critical role in the urine-concentrating mechanism. Na^+^-Cl^−^ absorption by water-impermeable TAL dilutes the luminal fluid and drives the renal countercurrent multiplication system that generates the axial osmolality gradient in the outer medulla, thereby facilitating the absorption of water by the CD (18). Mitochondria-poor principal cells may be able to withstand Gb3 accumulation and up-regulate *Aqp2* expression. Indeed, we found that they reacted to partially compensate for water loss caused by TAL dysfunction in *Gla^tm^Tg(CAG-A4GALT)* mice (Fig. 7*A*–*D*). Thus, water loss was not solely because of impaired CDs.

UMOD abundance in TAL, particularly in mTAL, was demonstrated to progressively decrease in Fabry disease (21). The level of UMOD expression was inversely proportional to the degree of lysosomal storage; it was suggested that abnormal UMOD expression in mTAL may contribute to the impaired urine-concentrating ability in Fabry disease. NKCC2 and UMOD in TAL contribute to the mechanism of polyuria. Loss-of-function mutations in *SLC12A1* in humans (44) and *Slc12a1* knockout in mice (46) lead to Bartter syndrome type I, and decreased levels of *Umod* are likely responsible for the polyuria observed in *Umod* knockout mice (47). We found that the down-regulation of 3 core molecules for Na^+^ reabsorption—Na^+^-K^+^-ATPase, UMOD, and NKCC2—contributed to the development of polyuria in *Gla^tm^Tg(CAG-A4GALT)* mice *via* impairment of TAL.

Gb3 is incorporated into the plasma membrane and intracellular membranes with a preference for lipid rafts (48). Alterations in lipid raft composition that were observed in Fabry disease induce changes in lipid raft dynamics (48). UMOD, NKCC2, and Na^+^-K^+^-ATPase are localized in lipid rafts. UMOD interacts with NKCC2 in apical trafficking (47, 49) and regulates the turnover, trafficking, and basolateral expression of Na^+^-K^+^-ATPase (50). TAL dysfunction in *Gla^tm^Tg(CAG-A4GALT)* mice may be caused, in part, by a decrease in these interactions.

The current study has several limitations. First, BP was not measured by the validated tail-cuff method (51), there was no training for the procedure, and fewer measurements were made. Moreover, compared with in-house mice, those mice that were obtained from vendors demonstrated a higher BP (52). Nonetheless, WT mice did not have high BP. Thus, BP values measured in this study may not differ significantly from those that would have been measured by the validated tail-cuff method. Overall, we believe that a difference in BP exists between *Gla^tm^Tg(CAG-A4GALT)* and WT mice. Second, although we evaluated NCC abundance by Western blot analysis, we did not examine the phosphorylation status of the protein (32), which is a more useful measure of activity.

Although tubular injuries have been observed early in the course of human Fabry disease (6, 9, 12–16, 41), it is unclear whether these injuries contribute to the development of end-stage renal disease. In *Gla^tm^Tg(CAG-A4GALT)* mice, mTAL injury that was directly caused by Gb3 accumulation, and not by podocyte injury, contributed to fibrosis, which led to renal dysfunction. It may therefore be important to note the occurrence of mTAL injury in addition to podocyte injury in human Fabry nephropathy.

In summary, we found that mTAL was the most severely affected tubule in our mouse model of Fabry disease with polyuria, and that TAL dysfunction—reduction in the levels of Na^+^-K^+^-ATPase, UMOD, and NKCC2—impairs the urine-concentrating ability. In addition, fibrosis associated with TAL impairment may be responsible for renal dysfunction. Results highlight the importance of mTAL dysfunction in the pathophysiology of Fabry nephropathy. Additional studies are required to verify whether our findings are relevant to the human disease and could provide a basis for the development of novel therapies.

## Supplementary Material

This article includes supplemental data. Please visit *http://www.fasebj.org* to obtain this information.

## Abbreviations

AQP2: aquaporin-2
AVP: arginine vasopressin
BP: blood pressure
CD: collecting duct
Cr: creatinine
DCT: distal convoluted tubule
FE: fractional excretion
Gb3: globotriaosylceramide
GLA: α-galactosidase A
IS: inner stripe
mTAL: medullary thick ascending limb
NCC: Na^+^-Cl^−^ cotransporter
NKCC2: Na^+^-K^+^-2Cl^−^ cotransporter
8-OHdG: 8-hydroxyl-2′-deoxyguanosine
OS: outer stripe
PAS: periodic acid–Schiff
Slc12: solute carrier family 12 member
TAL: thick ascending limb
UMOD: uromodulin
WT: wild type
