# Supplementary material for: Medullary thick ascending limb impairment in the GlatmTg(CAG-A4GALT) Fabry model mice
Source: FASEB J. 2018 Mar 19;32(8):4544–59. doi: 10.1096/fj.201701374R (PMC6071062; doi:10.1096/fj.201701374R)
Supplement: Supplementary file 1 [file fj.201701374R.st1.docx]

| **Antibody** | **Host** | **Source** | **Application (Reference^†^)** |
| --- | --- | --- | --- |
| **Anti-UMOD** | Sheep polyclonal | AbD Serotec  (8595-0054) | WB (S1), IHC (S2) |
| **Anti-NKCC2** | Rabbit polyclonal | Alpha Diagnostic  (NKCC21-A) | WB (S3) |
|  | Rabbit polyclonal | StressMarq Biosciences  (SPC-401D) | IHC (S1) |
| **Anti-Na^+^-K^+^-ATPase** | Rabbit polyclonal | Abcam  (ab76020) | WB (ND*), IHC (S4) |
| **Anti-F4/80** | Rat monoclonal | AbD Serotec  (MCA497GA) | IHC (S5) |
| **Anti-NCC** | Rabbit polyclonal | EMD Millipore  (AB3553) | WB (S6), IHC (S6) |
| **Anti-AQP2** | Rabbit polyclonal | Abcam  (ab110496) | WB (ND**) |
|  | Rabbit polyclonal | Abcam  (ab78230) | IHC (S7) |
| **Anti-AVPR2** | Rabbit polyclonal | Alomone Labs  (AVR-012) | WB (S8) |
| **Anti-MDA** | Mouse monoclonal | JaICA  (MMD-030n) | IHC (S9) |
| **Anti-GAPDH** | Rabbit polyclonal | Sigma-Aldrich  (G9545) | WB (ND*) |

SUPPLEMENTARY TABLE 1. *Validation of primary antibodies used for mouse western blotting and immunohistochemistry*

^†^References can be found in the Supplementary References.

*We searched articles in the PubMed database that had validated the antibodies but were unable to find any.

**Abcam sells six types of rabbit anti-AQP2 antibody but it is not known which of these was used since the article only mentions the company name.

IHC, immunohistochemistry; ND, not determined; WB, western blotting.
