# Supplementary material for: Medullary thick ascending limb impairment in the GlatmTg(CAG-A4GALT) Fabry model mice
Source: FASEB J. 2018 Mar 19;32(8):4544–59. doi: 10.1096/fj.201701374R (PMC6071062; doi:10.1096/fj.201701374R)
Supplement: Supplementary file 2 [file fj.201701374R.st2.docx]

SUPPLEMENTARY TABLE 2. *Validation of primary antibodies used for human immunohistochemistry*

| **Antibody** | **Host** | **Source** | **Application (Reference^†^)** |
| --- | --- | --- | --- |
| **Anti-UMOD** | Sheep polyclonal | AbD Serotec  (8595-0054) | IHC (S10) |
| **Anti-NKCC2** | Rabbit polyclonal | StressMarq Biosciences  (SPC-401D) | IHC (S11) |
| **Anti-Na^+^-K^+^-ATPase** | Rabbit polyclonal | Abcam  (ab76020) | IHC (S12) |
| **Anti-NCC** | Rabbit polyclonal | EMD Millipore  (AB3553) | IHC (S3) |
| **Anti-AQP2** | Rabbit polyclonal | Abcam  (ab78230) | IHC (ND*) |

^†^References can be found in the Supplementary References.

*We searched articles in the PubMed database that had validated the antibodies but were unable to find any.

IHC, immunohistochemistry; ND, not determined.
