# Supplementary material for: Medullary thick ascending limb impairment in the GlatmTg(CAG-A4GALT) Fabry model mice
Source: FASEB J. 2018 Mar 19;32(8):4544–59. doi: 10.1096/fj.201701374R (PMC6071062; doi:10.1096/fj.201701374R)
Supplement: Supplementary file 3 [file fj.201701374R.st3.docx]

SUPPLEMENTARY TABLE 3. *Clinical and laboratory data of patients with Fabry disease at the time of kidney biopsy*

| **Case** | **Sex** | **Age,**  **years** | ***GLA* mutation** | | **Type** | **CKD stage*** | **Biopsy indication** |
| --- | --- | --- | --- | --- | --- | --- | --- |
|  |  |  | **Nucleotide change** | **Amino acid change** |  |  |  |
| **A** | M | 49 | c.1228A>C | p.T410P | Classic | G2 | Proteinuria |
| **B (B2)** | F | 54 (69) | c.935A>G | p.Q312R | Late-onset | G1 (G2) | Proteinuria |
| **C (C2)** | M | 28 (40) | c.935A>G | p.Q312R | Late-onset | G1 (G2) | Proteinuria |
| **D (D2)** | F | 53 (65) | c.935A>G | p.Q312R | Late-onset | G1 (G3a) | Proteinuria |
| **E** | M | 26 | c.323_324insCAGA | p.A108fs | Classic | G1 | Proteinuria |
| **F** | F | 46 | c.1228A>C | p.T410P | Classic | G1 | Proteinuria |
| **G** | M | 47 | c.[1160T>C;1161C>T] | p.L387P | Classic | G2 | Proteinuria |
| **H** | M | 45 | c.[1160T>C;1161C>T] | p.L387P | Classic | G2 | Proteinuria |
| **I** | F | 33 | c.1244T>C | p.L415P | Classic | G1 | Proteinuria |

*Glomerular filtration rate category (ml/min/1.73 m^2^): G1, ≥ 90; G2, 60–89; G3a, 45–59.^S13^

CKD, chronic kidney disease; *GLA*, α-galactosidase A gene.
