# Supplementary material for: Medullary thick ascending limb impairment in the GlatmTg(CAG-A4GALT) Fabry model mice
Source: FASEB J. 2018 Mar 19;32(8):4544–59. doi: 10.1096/fj.201701374R (PMC6071062; doi:10.1096/fj.201701374R)
Supplement: Supplementary file 4 [file fj.201701374R.st4.docx]

SUPPLEMENTARY TABLE 4. *Clinical and laboratory data of patients with non-Fabry chronic kidney disease at the time of kidney biopsy*

| **Case** | **Sex** | **Age, years** | **Kidney disease** | **CKD stage*** | **Biopsy indication** |
| --- | --- | --- | --- | --- | --- |
| **1** | F | 21 | Minor glomerular abnormality | G1 | Hematuria |
| **2** | F | 26 | Minor glomerular abnormality | G2 | Proteinuria, hematuria |
| **3** | M | 42 | Minor glomerular abnormality | G1 | Proteinuria |

*Glomerular filtration rate category (ml/min/1.73 m^2^): G1, ≥ 90; G2, 60–89.^S13^

CKD, chronic kidney disease.
