# Supplementary material for: Medullary thick ascending limb impairment in the GlatmTg(CAG-A4GALT) Fabry model mice
Source: FASEB J. 2018 Mar 19;32(8):4544–59. doi: 10.1096/fj.201701374R (PMC6071062; doi:10.1096/fj.201701374R)
Supplement: Supplementary file 5 [file fj.201701374R.sd1.docx]

**Supplementary References**

1. El-Achkar, T. M., McCracken, R., Liu, Y., Heitmeier, M. R., Bourgeois, S., Ryerse, J., and Wu, X. R. (2013) Tamm-Horsfall protein translocates to the basolateral domain of thick ascending limbs, interstitium, and circulation during recovery from acute kidney injury. *Am. J. Physiol. Renal Physiol.* **304**, F1066–F1075
2. Nevo, N., Chol, M., Bailleux, A., Kalatzis, V., Morisset, L., Devuyst, O., Gubler, M. C., and Antignac, C. (2010) Renal phenotype of the cystinosis mouse model is dependent upon genetic background. *Nephrol. Dial. Transplant.* **25**, 1059–1066
3. Yang, S. S., Lo, Y. F., Yu, I. S., Lin, S. W., Chang, T. H., Hsu, Y. J., Chao, T. K., Sytwu, H. K., Uchida, S., Sasaki, S., and Lin, S. H. (2010) Generation and analysis of the thiazide-sensitive Na+ -Cl− cotransporter (Ncc/Slc12a3) Ser707X knockin mouse as a model of Gitelman syndrome. *Hum. Mutat.* **31**, 1304–1315
4. Dumková, J., Smutná, T., Vrlíková, L., Le Coustumer, P., Večeřa, Z., Dočekal, B., Mikuška, P., Čapka, L., Fictum, P., Hampl, A., and Buchtová, M. (2017) Sub-chronic inhalation of lead oxide nanoparticles revealed their broad distribution and tissue-specific subcellular localization in target organs. *Part. Fibre. Toxicol.* **14**, 55
5. Nagase, M., Kurihara, H., Aiba, A., Young, M. J., and Sakai, T. (2016) Deletion of Rac1GTPase in the myeloid lineage protects against inflammation-mediated kidney injury in mice. *PLoS One*. **11**, e0150886
6. Hunter, R. W., Ivy, J. R., Flatman, P. W., Kenyon, C. J., Craigie, E., Mullins, L. J., Bailey, M. A., and Mullins, J. J. (2015) Hypertrophy in the distal convoluted tubule of an 11β-hydroxysteroid dehydrogenase type 2 knockout model. *J. Am. Soc. Nephrol.* **26**, 1537–1548
7. Lian, P., Li, A., Li, Y., Liu, H., Liang, D., Hu, B., Lin, D., Jiang, T., Moeckel, G., Qin, D., and Wu, G. (2014) Loss of polycystin-1 inhibits Bicc1 expression during mouse development. *PLoS One*. **9**, e88816
8. Mamenko, M., Dhande, I., Tomilin, V., Zaika, O., Boukelmoune, N., Zhu, Y., Gonzalez-Garay, M. L., Pochynyuk, O., and Doris, P. A. (2016) Defective Store-Operated Calcium Entry Causes Partial Nephrogenic Diabetes Insipidus. *J. Am. Soc. Nephrol.* **27**, 2035–2048
9. Hirose, M., Yasui, T., Okada, A., Hamamoto, S., Shimizu, H., Itoh, Y., Tozawa, K., and Kohri, K. (2010) Renal tubular epithelial cell injury and oxidative stress induce calcium oxalate crystal formation in mouse kidney. *Int. J. Urol*. **17**, 83–92
10. Saito, S., Murakumo, Y., Tsuzuki, T., Dambara, A., Kato, T., Enomoto, A., Asai, N., Maruyama, S., Matsuo, S., and Takahashi, M. (2011) Analysis of glial cell line-derived neurotrophic factor-inducible zinc finger protein 1 expression in human diseased kidney. *Hum. Pathol.* **42**, 848–858
11. Zhuang, Y., Jia, Z., Hu, C., Ding, G., Zhang, X., Zhang, Y., Yang, G., Rohatgi, R., Huang, S., He, J. C., and Zhang, A. (2016) Albuminuria confers renal resistance to loop diuretics via the stimulation of NLRP3 inflammasome/prostaglandin signaling in thick ascending limb. *Oncotarget*. **8**, 75808–75821
12. Hoppensack, A., Kazanecki, C. C., Colter, D., Gosiewska, A., Schanz, J., Walles, H., and Schenke-Layland, K. (2014) A human in vitro model that mimics the renal proximal tubule. *Tissue Eng. Part C Methods*. **20**, 599–609
13. KDIGO 2012 clinical practice guideline for the evaluation and management of chronic kidney disease. (2013) *Kidney Int. Suppl.* **3**, S1–S150
